# ‘Why do an MPH?’ Motivations and intentions of physicians undertaking postgraduate public health training at the University of Cape Town

**DOI:** 10.3402/gha.v9.32735

**Published:** 2016-10-13

**Authors:** Virginia E.M. Zweigenthal, Emma Marquez, Leslie London

**Affiliations:** 1 School of Public Health and Family Medicine, Faculty of Health Sciences, University of Cape Town, Cape Town, South Africa; 2 Formerly an international exchange scholar at Mount Sinai School of Medicine, Columbia University, New York, NY, USA

**Keywords:** public health, public health education, medical education, South Africa

## Abstract

**Background:**

Public health (PH) approaches underpin the management and transformation of health systems in low- and middle-income countries. Despite the Master of Public Health (MPH) rarely being a prerequisite for health service employment in South Africa, many physicians pursue MPH qualifications.

**Objectives:**

This study identifies their motivations and career intentions and explored MPH programme strengths and gaps in under- and post-graduate PH training.

**Design:**

A cross-sectional study using an online questionnaire was completed by physicians graduating with an MPH between 2000 and 2009 and those enrolled in the programme in 2010 at the University of Cape Town.

**Results:**

Nearly a quarter of MPH students were physicians. Of the 65 contactable physicians, 48% responded. They were mid-career physicians who wished to obtain research training (55%), who wished to gain broader perspectives on health (32%), and who used the MPH to advance careers (90%) as researchers, policy-makers, or managers. The MPH widened professional opportunities, with 62% changing jobs. They believed that inadequate undergraduate exposure should be remedied by applying PH approaches to clinical problems in community settings, which would increase the attractiveness of postgraduate PH training.

**Conclusions:**

The MPH allows physicians to transition from pure clinical to research, policy and/or management work, preparing them to innovate changes for effective health systems, responsive to the health needs of populations. Limited local job options and incentives are important constraining factors. Advocacy for positions requiring qualifications and benchmarking exit competencies of programmes nationally may promote enrolment.

## Introduction

There has been a global call for educators to train health professionals to engage in patient-centred and population-orientated health care, who could then contribute to reduce health inequities and improve access to health services (1). In South Africa, regulations for medical training foreground public health (PH) in curricula (2). However, teaching time for PH is widely recognized as very limited in South Africa, with <8% of the curriculum allocated for PH across institutions. Professional and expert PH practice requires training at a postgraduate level.

Since 1994, South Africa's health system has undergone legislative and organisational restructuring from a fragmented, inequitable, hospicentric health service under apartheid to a primary healthcare-orientated, unified health service based on a district health system (3). Resources were redistributed geographically and between levels of care in an attempt to redress racial and other inequalities in health. Early post-apartheid human resources for health policy documents focused on clinical personnel without addressing the PH workforce (4). The 2011 Policy on Human Resources for Health in South Africa discusses the need for PH-trained personnel but is silent on training requirements such as postgraduate PH qualifications (5).

Master of Public Health (MPH) degrees have been introduced over the last two decades by eight universities in South Africa, attracting students with diverse undergraduate degrees in health, science, and social sciences. Courses were developed to create a workforce capable of improving health and healthcare in the region (6).

University of Cape Town's (UCT) MPH began in 1999, building on a prior MPhil degree in epidemiology. From inception to the end of 2010, 301 MPH students have enrolled in the course, of whom 70 were physicians (23%). Enrolment increased annually from classes of <10 to intakes of over 60 in recent years.

This programme, requiring on-site class attendance, offers a general track alongside, speciality tracks in epidemiology, health economics, clinical research (2009), and most recently, health systems research (2011) (7). Except for health economics which requires eight, all streams require completion of 10 half-year courses and a mini-dissertation, together totalling 1,800 h of work. For most tracks, core courses include epidemiology, biostatistics, research methods and ‘health and society’. Coursework comprises two-third of course credits and the balance is generated from a mini-dissertation, usually a piece of research written up as a publication-ready manuscript.

A skilled PH workforce is critical for the improvement of health status (8). PH expertise, through appropriate surveillance, analysis, policy-making, programming, and implementation, can improve health status in low- and middle-income countries (LMICs) (9–11). However, human resources shortages in healthcare, including the PH workforce, are a global challenge (12, 13). It has been argued in Africa (9) and India (14) that the MPH qualification should be a prerequisite for professional PH practice because PH competencies – particularly planning, data management, and community need assessments – are core to the work of district managers (15). But in many LMICs, including South Africa, there is no such requirement and a disconnect exists between an articulated need for PH skills and available service positions. Limited career prospects may deter PH training (14) and prompt migration out of the public sector (9).

Published research on motivations for PH training emanates from high-income countries (16–18). Currently, little is known about physicians’ motivations for postgraduate PH education in LMIC countries.

This study describes the motivations of medical physicians completing the MPH degree at a South African university, from the qualification's inception in 1999 until 2010, and their reflections on the value of this training for their subsequent careers. The experience in this programme may be useful to better understand the motivations and career paths opened by the training; the potential work niches for medically trained MPH graduates; and skills required for health systems in LMICs.

## Methods

In 2011, we conducted a cross-sectional analytic study amongst physicians completing MPH degrees between 2001 and 2010 or enrolled in the MPH programme at the UCT in 2010.

An online, self-administered, semi-structured questionnaire, created through the assessment module of the online student learning software, Sakai, was made available to respondents after being invited by email to the site address to participate in the study. Thirty-seven closed- and open-ended questions gathered information about demographics, studies, and careers; perspectives about the value of training; their PH training as medical students; and career intentions. Data were extracted from questionnaires and entered into an Excel spreadsheet.

To be enrolled in the study, participants had to 1) be registered for the MPH at UCT between 1999 and 2010; 2) be qualified physicians at MPH enrolment; and 3) have graduated before 2010, or remain enrolled in the MPH in 2010.

Prospective participants were identified from the MPH student database and university-held records. Physicians formed about 25% of each MPH cohort between 1999 and 2010 [interquartile range (IQR):15–27%] and comprised 70 (23%) of the 301 MPH students overall. As contact details were missing for 5, 65 physicians were invited to participate in an online survey and 31 responded. Non-responders were contacted twice by email, to encourage participation. Quantitative data analysis for variables derived from closed-ended questions was done using STATA 13. Descriptive analyses and summary statistics are reported for normal and non-normally distributed data. Differences in responses by demographic and training variables were explored using *t*-tests (to compare means), Mann–Whitney *U*-tests (to compare medians for non-normal data), chi-squared tests (for categorical data such as proportions), and Spearman's correlations (for numerical, non-normal distributed data) for hypothesis testing. Possible selection bias due to non-response of potential informants was explored by comparing demographic and educational variables of respondents to *all* physicians on the ‘MPH doctor database’ using Wilcoxon signed-rank tests for continuous non-normally distributed data, and z-score tests for categorical data. Levels of significance were *p*<0.05.

Inductive analysis, grouping responses into themes (19), was performed manually and independently by two authors (EM and VZ) on the qualitative information elicited from open-ended questions. Quotations best illustrating themes were selected. Respondents are identified by gender (‘M’ being male and ‘F’ being female) and by age in years, namely gender and age.

Participation in the study was voluntary; participants were assured of anonymity and no identifiers were captured. Respondents completed consent forms before proceeding to the questionnaire. Ethical approval for the study was obtained from the UCT Research Ethics Committee (HREC Ref.: 251/2010).

## Results

Of the 65 physicians invited to participate, 31 (48%) responded. The response rate did not differ by year of enrolment. Of the 31 respondents, 8 (25%) were enrolled in the MPH in 2010.

**Fig. 1:**
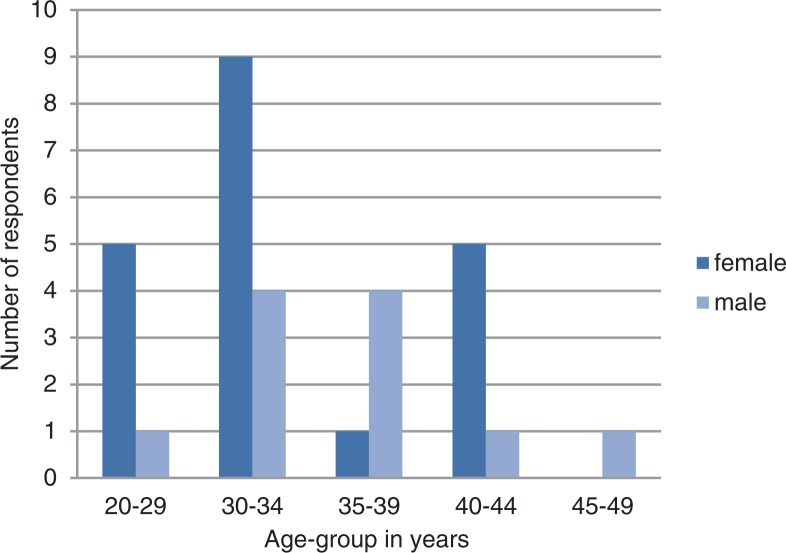
Age and gender of respondents (n=31).

The median age of the 31 participants at commencement of MPH studies was 33 years (IQR: 30–38 years). Twenty (65%) were female, a significantly higher proportion than male respondents (*p*=0.00). Although men were slightly older than women (median 35 years; IQR: 31–37 vs. median 32 years; IQR: 29–39.5), this difference was not statistically significant (Fig. 1).

Most (61%) participants were South African, 29% came from Anglophone African countries and 10% were from the USA. Foreign students entering the programme were significantly younger than South African students (mean age=30 vs. 36.5 years; *p*=0.00).

All completed medical degrees in their country of origin, except two Botswanans who trained before the establishment of a Botswanan school. Most (63%) South Africans had graduated in medicine at UCT. Besides medical degrees, 72% (21/29) held a range of degrees (from bachelors to masters) or clinical diplomas, and 45% had additional postgraduate qualifications including medical specialist qualifications.

Prior to commencing the MPH, most (71%) worked as non-specialist clinicians, of whom 36% worked at primary care level. Others were medical specialists (13%), specialists-in-training (13%), researchers (13%), or managers (6%). Before starting the MPH, 59% worked in government health services, 16% for private health institutions, and 6% worked at universities and research institutions.

Respondents’ roles within organisations are described in Table 1. All specialists and most clinicians worked in the state sector. Half the researchers worked for research institutions and a quarter each worked for the Department of Health and universities. Both managers worked in the private sector.

**Table 1:** Roles of MPH students – by employers prior to studying

	PHC clinician (n=8)	Clinician (n=7)	Specialist (n=4)	Registrar (n=4)	Researcher (n=4)	Manager (n=2)	Academic (n=1)	Student (n=1)
Government hospital (n=14)	5 (63%)	5 (71%)	3 (75%)	1 (25%)				
Dept of health (n=5)			1 (25%)	2 (50%)	1(25%)		1 (100%)	
University (n=3)		1 (14%)		1 (25%)	1 (25%)			
Research institution (n=2)					2 (50%)			
Private health provider (n=4)	2 (25%)					2 (100%)		
NGO (n=2)	1 (13%)	1 (14%)						
None (n=1)								1 (100%)
Total	(100%)	(100%)	(100%)	(100%)	(100%)	(100%)	(100%)	(100%)

### MPH programme selection

Compared to 50% of MPH students overall, significantly more (65%) respondents chose specialty tracks within the MPH programme (*p*=0.03). All four specialists took the clinical research track (started in 2009), and both managers completed the health economics track.

Respondents reported substantial work experience, and the mean interval from completing medical training to commencing MPH studies was 8.7 years (SD=5.3; range 1–20). Intervals have shortened significantly with each subsequent medical student cohort (ρ=−0.42; *p*=0.02). However, this finding may be confounded because the MPH only commenced in 1999.

Compared to part-time students, full-time students (*n*=13; 41%) were more likely to have moved to Cape Town to study (92.3% vs. 5.6%; *p*=0.00); be foreign (83.3% vs. 23.1%; *p*=0.001); be younger when starting studies (mean age=30.9; SD=4.41 vs. 36.0; SD=5.03; *p*=0.003); and not have other postgraduate qualifications (30.8% vs. 72.2%; *p*=0.03). Although not statistically significant, full-time students were more likely to have taken the Health Economics track (41.7% vs. 12.5%; *p*=0.1). No managers, academics, or specialists relocated in order to study, whereas 60% (9/15) of non-specialist clinicians relocated to study full-time.

Reasons given for selecting UCT were the location of the course (61%), its reputation (48%), and being the students’ alma mater (23%).

### Motivations for studying

Motivations for studying did not differ between younger and older respondents (≤median cf. >median age). A common motivation (55% overall and 65% for the clinical or epidemiology tracks) was the research training the MPH provided – skills to critically review research publications and to design and conduct research in clinical settings. Sixteen (52%) desired a career change. Some wanted to transition from pure clinical work to careers in clinical research or management. Half volunteered that PH perspectives and skills would enable them to work at a population level. Four (13%) younger students (less than the median age) reported that they decided on the degree as medical students, and they selected the health economics or epidemiology tracks.

### Career paths

**Fig. 2:**
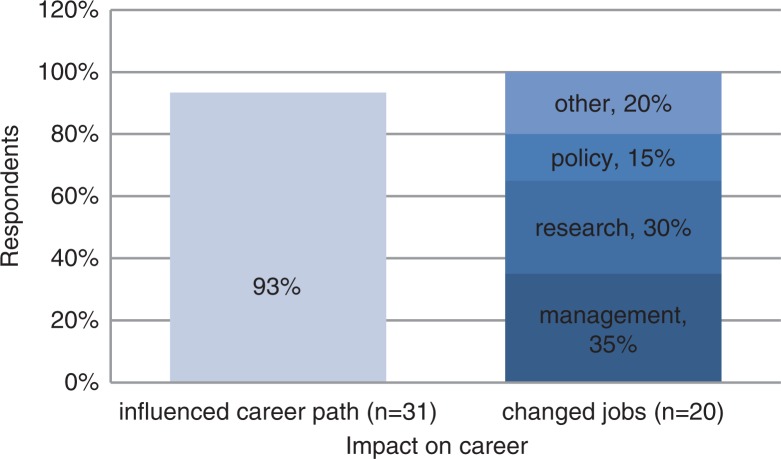
Impact of MPH on subsequent careers.

The MPH was used to develop careers and opened up work opportunities (Fig. 2). It influenced career directions for 90%, and 65% subsequently changed jobs. For 40%, the qualification facilitated research or policy work for which respondents would otherwise not have been qualified:

I would not be working in the areas I do right now without my MPH. The MPH opened up a lot of opportunities. (M, 33)

**Fig. 3:**
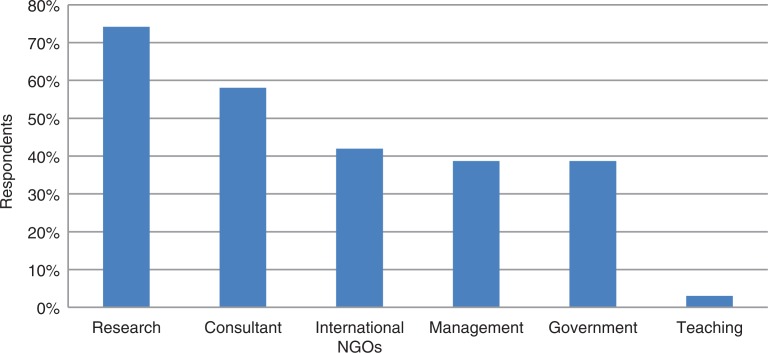
Possible career paths for MPH graduate physicians.

As is depicted in Fig. 3, respondents’ perceived value of the degree mirrored their experience and motivations. Overwhelmingly, research was seen as a career option (74%), followed by consultancy (58%), and international NGO work (42%). Government or management positions were identified by a minority (39%). Useful research skills volunteered were understanding the research process, interpreting findings (32%), and conducting research (19%).

Some believed the MPH could further physicians’ careers and others felt not having a postgraduate degree was career-limiting. The MPH could open doors to employment opportunities, allowing physicians to work in clinical research or management:

[It] enables those of us who have chosen not to specialise, to have an additional qualification … to remain in the health sector and advance into managerial positions. (F, 35)

Ten (32%) thought that broader, socio-ecological perspectives on illness gained through the MPH allowed physicians to contextualise clinical work and understand how social determinants of health, community factors, prevention, and health promotion impact on health outcomes. A quarter noted that the MPH enabled learning about health systems, policy, and resource allocation. A few (13%; *n*=4) remarked that skills learnt could equip physicians to become strategic managers.

### PH training as medical students

Five reported no exposure, and most had only a cursory introduction to PH as medical students. Twenty (65%) completed ‘stand-alone’ PH courses that were not integrated with clinical learning. Courses generally lasted 4 weeks but ranged from 2 weeks to 4 months. Few (23%) recalled PH content of these courses. Thirteen (42%) completed assigned PH projects including group research, health promotion, or short-term community attachments. Five (16%) had community-based volunteer experiences.

For many, this training was largely uninteresting. However, for a third, the exposure sparked postgraduate PH training. Non-South Africans were 4.9 times more likely to report a positive influence from their undergraduate PH exposure (95% CI: 1.95–12.2; *p*=0.00).

Nonetheless, many believed tailored, medical curricular PH exposure would attract physicians to postgraduate PH studies and made suggestions for its focus, learning settings and content (Table 2). Many argued that exposure should include community attachments and the integration of PH learning in clinical contexts – highlighting PH issues relevant to clinical practice; emphasising synergies to make the value of PH explicit. For example, the use of PH constructs, such as ‘levels of prevention’ and ‘social determinants of health’, assists in developing upstream interventions for disease prevention:

Medical students need to be taught that health does not start in the hospitals but at home and in communities. Something needs to be done even before the patients come to us as opposed to waiting for them and treating them in the hospital and then forgetting about them. (F, 28)

Public health should not be portrayed as at odds with clinical medicine. It should be portrayed as something strengthening it. (M, 36)

**Table 2:** Suggested focuses of public health curricula in medical training

Rationale for teaching	Setting for learning
Role of public health	Community attachments
Career options	Integrate and insert into clinical context
Market public health	

Content	

Overview	Biostatistics
What is public health?	Data analysis
Health systems	Health economics
Health services	Financing of health systems
Epidemiology	Cost-effectiveness
Burden of disease	Resource utilisation and rationing
Role in policy making, health management Monitoring and evaluation	Other
Research	Environmental health and climate change
Critical appraisal of literature	Occupational health
Study design	Health promotion
	Management and leadership

Over half believed that exposure to epidemiology, biostatistics, monitoring and evaluation, critical appraisal of journal articles, and evidence-based medicine were important skills and would attract medical students to postgraduate PH training. Other suggestions included being taught about PH's role in health systems, particularly in management and policy-making and about cost-efficient clinical practice in resource-constrained health service environments. A few believed that they should learn how to conduct PH research and highlighted its role in the promotion of health in communities:

[Medical students should acquire a] basic understanding of the benefits and thinking behind evidence-based medicine, critical appraisal, reading medical literature with basic understanding of research design and statistical concepts. By the end of medical school I still didn't understand what a *p*-value or 95% CI was. (M, 37)

Most remarked (65%) that PH training options were not profiled during medical training. Some suggested that being explicit about PH career options could encourage PH training, and suggested mentors and alumni speak and write about their career trajectories.

### Improving the MPH programme

Suggestions for programme improvement centred on accrediting the MPH and developing apprenticeships for MPH students. Respondents queried the advantage of the MPH qualification in the context of limited job options in the state health sector:

It is not a career advantage [in the public health system currently in South Africa], as there are very few posts for graduates, and most [are] at a lower pay level. (F, 52)

Others argued that a practical experience in service settings, applying theory learnt, would give insight into possible careers:

[An] ‘apprenticeship’ could be attractive – either during or after the degree. This would give a more realistic picture of what types of work is being done in the field. (F, 52)

## Discussion

Nearly a quarter of UCT's MPH students were physicians, lower than the 51% reported in the African Field Epidemiology Training Programme (20). The lower proportions of physicians in South Africa may result from the newness of the degree in South Africa, lack of professional prominence amongst physicians or limited financial advantage, as it is rarely a requirement for a public sector post.

The predominance of female physicians completing MPHs is consistent with local and international trends. Most (56%) medical students in South Africa over a similar period were women (21) and the temporal trend towards females mirrors national and international trends for registered medical practitioners in South Africa (21).

Overall, they were a mature group of experienced professionals and many were mid-career, as was also found in a 1998–1999 review of 40 accredited American graduate PH programmes where the typical enrolee was a mid-career professional studying part-time (22). Work and life experience may draw physicians into local PH programmes that prepare them for research, management, or policy work.

Interestingly, younger, non-South African, and more recently qualified physicians embarked on MPH studies sooner after graduation than physicians who qualified longer ago. They studied full-time, relocated for studies, and were foreign, using the MPH as a spring-board to change from clinical to research, policy, or management work. These physicians may experience pressure to obtain additional qualifications for career advancement.

Respondents’ career selections are reported elsewhere in the literature: graduates from Ugandan PH programmes became managers (23); Tulane university MPH graduates undertook population-oriented research (18); and many USA MPH graduates perform policy work in the Food and Drug Administration or National Institutes of Health (24).

The course seems to be an asset for health systems development in Africa with more than a quarter of respondents coming from Africa and 30% moving to Cape Town to pursue studies full-time. Funding to develop a cadre of African health economists facilitated the relocation of students to Cape Town for the flagship health economics track. Candidates were required to return to their home country, but it is not known what proportion of the 71% who moved for this track, complied.

Important motivations for training included developing research and epidemiological skills, and epidemiology is an acknowledged core strength of UCT's School of PH. Reviews of high income countries (HIC) graduate PH programmes found that population approaches to health issues and healthcare organisation were particularly valued. For example, Oxford University's global health programme graduates desired perspectives on social determinants of health (25), and Tulane MPH-MD graduates believed the MPH enabled the delivery of comprehensive individual and community care (18, 26). These perspectives have since become core to current undergraduate PH competencies in South Africa, but may not have been prominent when these respondents were undergraduates.

The UCT MPH does not have a particular focus on management training, which may explain why few desired management training, or perceived that the MPH was suited to physicians wanting such training. High-level management training may be more suited to a teaching model that emphasises experiential learning.

Even though postgraduate PH training in South Africa is generally not a requirement for health management, respondents reported that the MPH enabled careers in health services. This may reflect the informal value given to the MPH by employers, which accords with respondents’ perceptions that the MPH opened employment opportunities they would not otherwise have had.

The reported poor exposure and preparation to take on PH challenges as medical students was also found in a large British study about PH specialist training (27). There is some controversy about the appropriateness of detailed PH learning at undergraduate level (28). Some believe that medical students are not mature enough to grasp or appreciate population perspectives, approaches, and tools (29). Current health sciences educators, however, argue that training should prepare physicians to understand, work in, and transform systems using skills that address inequities in health status (1), a perspective firmly within the domain of PH.

Medical school exposure appeared to impact more on the foreign graduates’ decisions to study PH than on South African physicians. This may be the result of an inadequate PH undergraduate curriculum for South African medical students when they studied, as well as recall bias from an older cohort of physicians. Medical education curriculum changes implemented in many South African universities in the early 2000s have resulted in PH becoming more central in medical teaching (30). This may attract young and recent graduates to PH training.

For those whom medical school exposure was a motivator for studying PH, a ‘hands-on’ community experience was formative and may attract physicians to PH training and careers. A USA study also found that physicians, participating in an 8-week PH undergraduate field experience in developing countries, were more likely than their counterparts to subsequently practice primary care and obtain advanced PH degrees (31). An experiential community-based focus has been identified as core to medical education reform by the working group of undergraduate South African medical PH educators (32).

Modules, with varying teaching models and course content, could address the learning needs of both mid- and early-career physicians. Findings revealed common and discrete learning needs, and coursework covering core PH disciplines with focused electives, using both teacher- and student-driven approaches and assessment, could be tailored to learning needs.

The suggested creation of practical service exposures accords with calls to embed these experiences into PH professional training (33), which should prepare and attract recent graduates to health service work. Optional service attachments – ‘practica’ – allowing students to work alongside service providers have been introduced at UCT, as an elective, and are core to the Ugandan (23) and most accredited USA MPH programmes (13).

The need to benchmark MPH programmes raised by respondents is in line with African and international trends, which call for quality assured degrees confirming core competencies (34, 35). Core compulsory courses in PH disciplines together with elective modules could lead to MPHs becoming ‘a professional degree whose primary purpose is to prepare students for PH practice’ as was the case in Canada (36).

MPH graduates working in health settings could help ensure health system responsiveness to major burdens of disease and better use of data. This would guide decision-making and improve effectiveness and efficiency, enhancing health system resilience.

### Study limitations

A limitation of the study is the small sample size and 48% response rate. However, respondents did not differ from all physicians completing MPHs at UCT in terms of gender, nationality, MPH cohort, year of qualification as physicians, and age commencing postgraduate PH studies. This suggests that respondents are representative of the general UCT MPH doctor population. Nonetheless, respondents may value the MPH course more highly than non-respondents.

The motivations of physicians undertaking the MPH at UCT may not be generalisable to later student cohorts and to other South African programmes, as UCT MPH's strengths are health economics and epidemiological research, which may attract physicians interested in these areas. Recent changes in UCT course offerings, responding to changes in the South African policy environment, may attract physicians with other interests.

The perception that medical student PH exposure is inadequate may be due to memory attrition or enthusiastic PH physicians judging a necessarily limited undergraduate curriculum harshly.

Graduates from other disciplinary backgrounds were not included and it is not known if their motivations and career paths differ from physicians. Comparative studies comparing PH graduates from various backgrounds and South African universities are a fruitful area for future research.

## Conclusion

This study fills a gap in international and local PH education research. There is scant literature focussed on the motivations and career intentions of professionals embarking on post-graduate PH training, yet such knowledge is important to attract quality candidates and to build a competent PH workforce. Physicians, a substantial proportion of UCT's MPH students, traditionally undertake PH studies mid-career but recent graduates sought PH training earlier in their careers. This shift is encouraging and may reflect an increasing realisation of the importance of, exposure to, and contribution of PH to health systems.

The preponderance of women enrolled accords with the feminisation of Medicine nationally and internationally, and challenges employers to create working environments that attract younger women physicians.

The poor impact of prior PH education on physicians’ motivations for postgraduate training challenges educators to expose medical students to settings that demonstrate PH's value, roles for physicians, which, in turn, will attract physicians to PH practice. The impact of such exposure is a fruitful area for research.

In our study, physicians undertaking MPHs seek career changes, skills development, and job promotion to contribute to equitable and responsive health systems, moving away from clinical medicine. The course exposed physicians to population perspectives on health and illness, which informed their day-to-day work and job choices.

The MPH is an important programme to build skills and human resources for health system reform. Graduates can innovate changes required for an effective health system such as envisaged by South Africa's National Health Insurance scheme (37).

Training institutions should consider advocacy for a range of service positions – technical and managerial, for the growing number of graduates with postgraduate PH qualifications. The MPH may require bench-marking to assure competency, through training institutions agreeing about core competencies, to assure the quality of graduates to meet the health needs of populations.

## Paper context

A skilled public health workforce is required to transform health systems in low- and middle-income countries. Although postgraduate public health training is rarely required for posts in the South African health system, many physicians undertake MPH training. Reasons include a desire for skills to address health inequities and career transition to research, policy, and/or management. Advocacy for incentives and career opportunities linked to MPH qualifications based on benchmarked programme exit competencies is required.
